# Multiparameter Analysis of Gas Transport Phenomena in Shale Gas Reservoirs: Apparent Permeability Characterization

**DOI:** 10.1038/s41598-018-20949-2

**Published:** 2018-02-08

**Authors:** Yinghao Shen, Yu Pang, Ziqi Shen, Yuanyuan Tian, Hongkui Ge

**Affiliations:** 1 Unconventional Natural Gas Institute, China University of Petroleum, Beijing, 102249 China; 2 Department of Petroleum Engineering, Texas Tech University, Lubbock, TX 79409 USA; 3 State Key Laboratory for Oil and Gas Reservoir Geology and Exploitation, Chengdu University of Technology, Chengdu, 610059 China; 4 China University of Petroleum-Beijing at Karamay, Karamay, 834000 China

## Abstract

The large amount of nanoscale pores in shale results in the inability to apply Darcy’s law. Moreover, the gas adsorption of shale increases the complexity of pore size characterization and thus decreases the accuracy of flow regime estimation. In this study, an apparent permeability model, which describes the adsorptive gas flow behavior in shale by considering the effects of gas adsorption, stress dependence, and non-Darcy flow, is proposed. The pore size distribution, methane adsorption capacity, pore compressibility, and matrix permeability of the Barnett and Eagle Ford shales are measured in the laboratory to determine the critical parameters of gas transport phenomena. The slip coefficients, tortuosity, and surface diffusivity are predicted via the regression analysis of the permeability data. The results indicate that the apparent permeability model, which considers second-order gas slippage, Knudsen diffusion, and surface diffusion, could describe the gas flow behavior in the transition flow regime for nanoporous shale. Second-order gas slippage and surface diffusion play key roles in the gas flow in nanopores for Knudsen numbers ranging from 0.18 to 0.5. Therefore, the gas adsorption and non-Darcy flow effects, which involve gas slippage, Knudsen diffusion, and surface diffusion, are indispensable parameters of the permeability model for shale.

## Introduction

**Figure 1:**
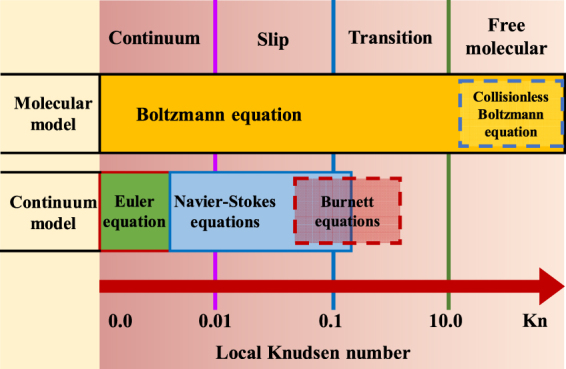
Gas flow regimes categorized by Knudsen number^16^.

Shale gas production is a major constituent of natural gas production in the United States. Although profitable shale gas production has been achieved using hydraulic fracturing and horizontal drilling technologies, several subjects are worth studying. First, shale is a good adsorbent of adsorptive gases, such as methane (*CH*_4_), carbon dioxide (*CO*_2_), and nitrogen (*N*_2_). Accordingly, free gas and adsorbed gas coexist in shale gas reservoirs^1–4^. Many experimental measurements of the gas adsorption capacity of shale rocks have been conducted to assess their adsorption mechanisms and to correct the original gas in place (*OGIP*) in shale gas reservoirs^5–7^. Second, shale is an ultralight rock. Its extremely low permeability and micro- and nanoscale pore sizes are very different than those of normal conventional rocks (sandstone and carbonate). Thus, Darcy’s law cannot be used to interpret the gas flow behavior in shale gas reservoirs^8–12^. In general, it is widely accepted that the flow regimes in micro- and nanoscale systems depend on the Knudsen number (*Kn*)^13–16^. As shown in Fig. 1, four flow regimes can be categorized based on their Knudsen numbers. For *Kn* < 0.001, the no-slip boundary condition in the continuum flow regime is valid, which indicates that Darcy’s law is applicable to this regime. For 0.001 < *Kn* < 0.1, the flow regime is the slip flow regime, which indicates that gas slippage occurs at the pore wall. At this time, the Navier-Stokes (N-S) equation should be solved under the slip boundary condition. For 0.1 < *Kn* < 10, the flow regime is a transition regime, which indicates that both gas slippage and gas diffusion occur simultaneously; thus, the slip model becomes more complex. Finally, for *Kn* > 10, free-molecule flow occurs, in which the intermolecular collisions are negligible compared to the collisions between gas molecules and pore walls. Third, surface diffusion may significantly contribute to mass transfer in porous media^17,18^. This diffusion coexists with the transfer of bulk free gas in shale, which is expressed by Fick’s law with surface diffusivity (*D*_*s*_). The mobility of adsorbed gas relative to that of bulk free gas determines the enhancement or impediment of surface diffusion with respect to gas transport in nanopores^19^. All of the abovementioned subjects are important for characterizing the properties of shale gas reservoirs. Therefore, the purpose of this study is to develop a model that includes multiple parameters to represent the unique features of shale rocks.

Some experimental and modeling studies have been conducted to investigate the apparent permeability of shale, as permeability is the most important parameter for predicting the gas production from shale gas reservoirs^8,9,19–24^. We summarize several representative apparent permeability models in Table 1.

**Table 1:** Summary of apparent permeability models for shale.

Model	Description
Javadpour^8^	Linear sum of first-order slip flow and Knudsen diffusion
Civan *et al*.^20^	A model that incorporates the suite of continuum, slip, transition, and free-molecular flow regimes in one equation and considers the gas adsorption/desorption effect
Darabi *et al*.^21^	Modified version of Javadpour’s model, with consideration of the impact of surface roughness on Knudsen diffusion
Moghadam and Chalaturnyk^22^	An expansion of the Klinkenberg slip theory expressed in quadratic format
Sheng *et al*.^23^	Nonlinear assembly of viscous flow and Knudsen diffusion associated with the surface diffusion effect
Wu *et al*.^9^	Nonlinear assembly of first-order slip flow and Knudsen diffusion associated with surface diffusion
Pang *et al*.^4,19,34^	Combination of second-order slip flow and surface diffusion using the Langmuir slip model, with consideration of the density profile provided by the SLD-PR model
Fink *et al*.^24^	Slip flow expressed in terms of the superposition of the Klinkenberg and pore-elastic effects

All of the proposed apparent permeability models involve non-Darcy flow corrections. The non-Darcy flow corrections represent different combinations of gas slippage, Knudsen diffusion, and surface diffusion. However, there are drawbacks that restrict the application of these apparent permeability models to estimate the permeability of shale gas reservoirs. The most remarkable limitation is that there are numerous variables in these apparent permeability models, such as gas slip coefficients, effective pore widths or pore diameters, surface diffusivity, and Klinkenberg correction factors. To determine these parameters, specific experimental measurements are required. Additionally, the main component of shale gas produced from shale gas reservoirs is methane, which is an adsorptive gas. Therefore, the reduction in the effective pore width or pore diameter due to the adsorption of gas molecules on the surfaces of pore walls should be considered, especially for nanoporous shale. Otherwise, the Knudsen number will be underestimated, which will impact the flow regime judgement.

Therefore, in this study, the requisite experimental measurements are conducted by assessing the Barnett and Eagle Ford shale outcrops to determine their pore size distribution, methane adsorption capacity, adsorption thickness, stress-dependent pore volume, and shale matrix permeability. The pore size distribution is obtained from nitrogen adsorption/desorption isotherms interpreted using the density functional theory (*DFT*) and Barrett-Joyner-Halenda (*BJH*) methods. The simplified local-density model associated with the Peng-Robinson EOS (*SLD-PR*) model, coupled with the pore size distribution, is used to describe and estimate the excess methane adsorption measured in the laboratory. In addition, the adsorption thickness is determined based on the density profile obtained using the SLD-PR model. The stress-dependent pore volume is measured via the gas expansion method; thus, the pore compressibility is calculated. Finally, the shale matrix permeability is measured using the pressure-pulse decay method. Subsequently, an apparent permeability model is developed that considers the effects of gas adsorption, stress dependence, and non-Darcy flow. The non-Darcy flow correction is expressed as a combination of gas slippage, Knudsen diffusion, and surface diffusion. Finally, the developed apparent permeability model is utilized to depict the measured shale matrix permeability when conducting regression analysis. The curve-fitting results validate the reliability of the developed apparent permeability model and reveal the importance of considering the effects of gas adsorption on the reduction in the effective pore width and the enhancement of gas transport in shale due to surface diffusion.

## Experimental Study

In this section, the experimental measurements of the methane adsorption capacity, pore size distribution, pore compressibility, and rock permeability of the Barnett and Eagle Ford shales are described.

### Samples

To conduct the previously mentioned experimental measurements, two shale core plugs were subsampled from a long outcrop of the Barnett Shale reservoir and two shale core plugs were subsampled from a long outcrop of the Eagle Ford Shale reservoir. For both the Barnett and Eagle Ford shale core plugs, one of them was analyzed to measure its permeability and then crushed to measure its total organic content (*TOC*), vitrinite reflectance (*R*_0_), and mineral components. The other was analyzed to measure its pore compressibility and then crushed to measure its nitrogen and methane adsorption capacity. The rock evaluation results and the mineral components of the Barnett shale and Eagle Ford shale outcrops are listed in Tables 2 and 3, respectively. Their total organic content (*TOC*) and vitrinite reflectance (*R*_0_) values were obtained from Rock-Eval pyrolysis. The rock mineral components of the whole-rock samples and clay minerals were determined via X-ray diffraction (*XRD*) analysis.

**Table 2:** Characteristics of the Barnett and Eagle Ford shale outcrops.

Rock Evaluation					
Core Sample	Bulk Density (g/cm^3^)	Grain Density (g/cm^3^)	TOC (wt%)	Kerogen Type	Vitrinite Reflectance R_o_ (%)
Barnett	2.304	2.608	12.87	II	0.45
Eagle Ford	2.327	2.761	4.82	II	0.72

**Table 3:** Mineral components of the Barnett and Eagle Ford shale outcrops.

Mineral Component (wt%)										
Core Sample	Quartz	Feldspar	Calcite	Dolomite	Pyrite	Smectite	Illite	Kaolinite	Chlorite	Illite/Smectite
Barnett	54	6	0	0	7	0	18	3	2	10
Eagle Ford	12	2	68	0	2	0	9	5	0	2

### Pore Size Distribution from Nitrogen Adsorption/Desorption Isotherms

**Figure 2:**
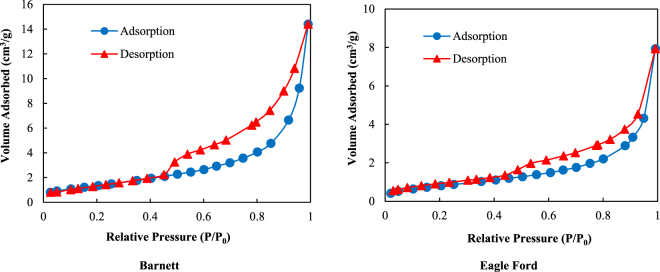
Nitrogen adsorption/desorption isotherms of the Barnett and Eagle Ford shale core samples.

**Figure 3:**
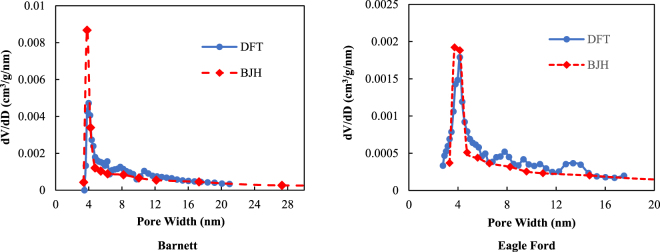
Pore size distribution based on the N_2_ adsorption/desorption isotherms obtained using the DFT and BJH methods.

The low-pressure nitrogen (*N*_2_) adsorption/desorption isotherms are measured to investigate the pore size distribution of the Barnett and Eagle Ford shale core samples. A commercial *N*_2_ adsorption/desorption pore size analyzer with a 0.0001 cm^3^/g (STP) minimum pore volume and pore sizes ranging from 0.35 to 400 nm is used in this study. For each of these two shale core samples, 1 g of each ground shale core sample (60 mesh) was dried and vacuumed overnight at 80 °C before conducting the *N*_2_ adsorption/desorption measurements. The *N*_2_ adsorption/desorption data are collected at 77 K (liquid nitrogen at atmospheric pressure). According to the IUPAC classification, the measured N_2_ adsorption/desorption isotherms for the Barnett and Eagle Ford shales exhibited in Fig. 2 are Type IV^25^. The hysteresis loop indicates the occurrence of capillary condensation in the mesopores^25,26^. The measured *N*_2_ isotherms are interpreted using both the density functional theory (*DFT*) and Barrett-Joyner-Halenda (*BJH*) methods. The pore size distributions are presented in Fig. 3. For the Barnett shale core sample, the mode of the pore widths obtained using DFT is 3.969 nm and that obtained using the BJH methods is 3.788. Thus, this Barnett shale core sample contains numerous mesopores with pore diameters of approximately 3.7 to 4 nm. For the Eagle Ford shale core sample, the mode of the pore widths obtained using DFT is 4.152 nm and that obtained using the BJH methods is 3.721. Similarly, this Eagle Ford shale core sample contains numerous mesopores with pore diameters of approximately 3.7 to 4.2 nm.

### Methane Adsorption Measurement

The methane adsorption capacity of the Barnett and Eagle Ford shale core samples is measured using the gravimetric method. In this study, non-adsorbed gas (helium) and adsorbed gas (methane) are used as injection gases to conduct two separate measurements for each of the Barnett and Eagle Ford shale core samples. The weight difference between the two measurements with different gases is regarded as the weight of the adsorbed gas. The selected experimental equipment is the magnetic suspension sorption system. The resolution of this apparatus is 0.01 mg, and its reproducibility is ±0.02 mg. The relative error is less than 0.002% of the measured value. To avoid the effects of the presence of moisture and formation water on the methane adsorption measurements^5,27,28^, the core sample was dried in a vacuum oven at 105 °C for 48 hours to remove the moisture and formation water before the adsorption measurements were conducted. Additional drying was performed by setting the core plug in the magnetic suspension sorption system at the testing temperature and vacuuming until the weight variation of the core sample was nil. In this study, high-purity (99.99%) methane (*CH*_4_) and high-purity (99.99%) helium (*He*) are utilized as injection gases, and the temperature of the adsorption measurements is set at 180 °F (82.22 °C) to mimic the reservoir conditions of the Barnett and Eagle Ford shales. Finally, the Gibbs (excess) adsorption isotherms of methane (*CH*_4_) for the Barnett and Eagle Ford shale core samples are determined, which are displayed in Section 4.

### Pore Compressibility

**Figure 4:**
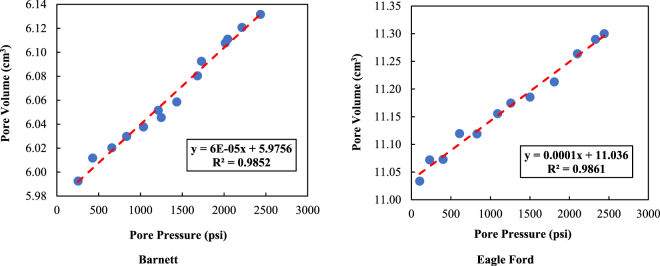
Pore volume versus pore pressure of the Barnett and Eagle Ford shale core samples measured via helium.

The gas expansion method is used to test the rock compressibility of the Barnett shale core sample. The experimental devices shown in Appendix A are applied to perform this test. In this study, a non-adsorptive gas (*He*) is chosen as the measuring gas. To mimic the reservoir conditions, the confining pressures in the axial and radial directions are set as 41.37 MPa (6000 psi) and 34.47 MPa (5000 psi), respectively. For more details on the pore compressibility measurements, please refer to the previous study^4^. The measurement results, which are expressed as pore volume versus pore pressure, are presented in Fig. 4.

### Permeability Measurement

A pressure-pulse decay permeability test was conducted, using the Autolab 1000 system, to determine the matrix permeability of the Barnett and Eagle Ford shale core samples. The precision of the measured permeability is approximately ±2.5%. The size and weight of the Barnett shale core sample are 1.5 × 3-inch and 200.182 g, respectively. Additionally, the size and weight of the Eagle Ford shale core sample are 1.5 × 3-inch and 202.163 g, respectively. Prior to testing the matrix permeability, the shale core sample was dried by placing it in a vacuum oven for 24 hours. To simulate the gas flow in shale gas reservoirs, high-purity methane (99.99%) is used as the measuring gas. During the permeability measurements, the confining pressure is set as a constant (15 MPa), and the matrix permeabilities with different pore pressures are measured. The shale matrix permeability versus the pore pressure of the Barnett shale and that of the Eagle Ford shale are presented in Section 4.

## Theory and Model Construction

In this section, the theories of gas adsorption, stress dependence, and non-Darcy flow applied in this study are presented. To precisely characterize the apparent permeability of shale, a permeability model that includes these critical parameters is constructed.

### Gas Adsorption Effect

A series of gas adsorption experimental measurements have been conducted to investigate the gas adsorption capacity of organic-rich shale^27,29–31^. Typically, organic matter (kerogen) and clay minerals are good adsorbents of methane (*CH*_4_), carbon dioxide (*CO*_2_), and nitrogen (*N*_2_)^27,32,33^. According to previous studies of gas adsorption in shale and coal, gas adsorption not only contributes to the original gas in place (*OGIP*) but also acts to reduce the hydraulic radius for gas transfer^4,7,34^. Herein, we focus on determining the thickness of adsorbed gas and analyzing the impact of adsorption on gas transport in nanopores.

Note that the thickness of adsorbed gas is related to the amount of adsorption, which can be calculated based on the density profile in the nanopores^34^. To determine the density distribution in the nanopores, the SLD-PR model is utilized to evaluate the methane adsorption performance of the Barnett and Eagle Ford shale core samples. The SLD-PR model, which considers the fluid-fluid and fluid-solid interactions in a slit-shaped pore, has been successfully used to describe the adsorption of gas on coal and shale rocks^30,35,36^. In the SLD-PR model, the equilibrium chemical potential is the sum of the potentials from fluid-fluid and fluid-solid interactions, which is equal to the bulk fluid potential.

1 $$\mu (z)={\mu }_{{\rm{ff}}}(z)+{\mu }_{{\rm{fs}}}(z)={\mu }_{{\rm{bulk}}}$$

where *µ(z)* is the chemical potential of fluid at position *z*. The subscripts “*bulk*”, “*ff*” and “*fs*” denote the bulk fluid, fluid-fluid and fluid-solid interactions, respectively.

The chemical potential of the bulk fluid can be written as a function of fugacity:

2 $${\mu }_{{\rm{bulk}}}={\mu }_{0}(T)+RT\,\mathrm{ln}(\frac{{f}_{{\rm{bulk}}}}{{f}_{0}})$$

Where the subscript “0” refers to an arbitrary reference state and *f*_bulk_ refers to the fugacity of the bulk fluid. Similarly, the chemical potential of a fluid-fluid interaction can be calculated using:

3 $${\mu }_{{\rm{ff}}}(z)={\mu }_{0}(T)+RT\,\mathrm{ln}(\frac{{f}_{{\rm{ff}}}(z)}{{f}_{0}})$$

where *f*_ff_*(z)* is the fugacity of the fluid-fluid interaction at position *z*.

The chemical potential of a fluid-solid interaction is given as:

4 $${\mu }_{{\rm{fs}}}(z)={N}_{A}[{{\rm{\Psi }}}^{{\rm{fs}}}(z)+{{\rm{\Psi }}}^{{\rm{fs}}}(L-z)]$$

where *N*_*A*_ is Avogadro’s number and *ψ*^*fs*^*(z)* and *ψ*^fs^(*L-z*) are potential energy functions that account for a fluid molecule at position *z* interacting with both slit walls. According to Lee’s partially integrated 10–4 Lennard-Jones potential^37^, the fluid-solid interaction can be calculated using the following equation:

5 $${\Psi }^{{\rm{fs}}}(z)=4\pi {\rho }_{{\rm{atoms}}}{\varepsilon }_{{\rm{fs}}}{\sigma }_{{\rm{fs}}}^{2}[\frac{{\sigma }_{{\rm{fs}}}^{10}}{5{(z^{\prime} )}^{10}}-\frac{1}{2}\sum _{i=1}^{4}\frac{{\sigma }_{{\rm{fs}}}^{4}}{{(z^{\prime} +(i+1){\sigma }_{{\rm{ss}}})}^{4}}]$$

In Equation 5, *ρ*_atoms_ is the solid atom density, which is equal to 38.2 atoms/nm^2^^36^. *ε*_fs_ is the fluid-solid interaction energy parameter. *σ*_fs_ is the average of *σ*_ff_ and *σ*_ss_, which is expressed as *σ*_fs_ = *(σ*_ff_ + *σ*_ss_*)/*2. *σ*_ff_ and σ_ss_ represent the molecular diameter of the adsorbate and the carbon interplanar distance, respectively. The value of the carbon interplanar distance is defined as that for graphite, i.e., 0.335 nm (*σ*_ss_ = 0.335 nm). *z′* is the dummy coordinate, which is defined as *z′* = *z* + *σ*_ss_/*2*.

By substituting Equations 2, 3 and 4 into Equation 1, the adsorption equilibrium can be expressed as:

6 $${f}_{{\rm{ff}}}(z)={f}_{{\rm{bulk}}}\cdot \exp (-\frac{{\Psi }^{fs}(z)+{\Psi }^{fs}(L-z)}{kT})$$

where *k* is Boltzmann’s constant (*k* = 1.38 × 10^−23^ J/K).

As mentioned above, the Peng-Robinson equation of state (PR-EOS) is applied to describe the fluid-fluid interaction. The PR-EOS can be written in terms of density as follows^36,38^:

7 $$\frac{p}{\rho RT}=\frac{1}{(1-\rho b)}-\frac{a(T)\rho }{RT[1+(1-\sqrt{2})\rho b][1+(1+\sqrt{2})\rho b]}$$

where

8 $$a(T)=\frac{0.457535\alpha (T){R}^{2}{T}_{c}^{2}}{{p}_{c}}$$

9 $$b=\frac{0.077796R{T}_{c}}{{p}_{c}}$$

In Equation 8, Gasem *et al*. (2001) introduced the term α(T), which is expressed as follows^39^:

10 $$\alpha (T)=\exp [(A+B{T}_{r})(1-{T}_{r}^{C+D\omega +E{\omega }^{2}}]$$

where *T*_*r*_ = *T* / *T*_*c*_ and *A*, *B*, *C*, *D* and *E* are correlation parameters with values of 2.0, 0.8145, 0.134, 0.508 and −0.0467, respectively. The value of the acentric factor ω is set as 0.0113 in this study. Methane is the only adsorbed gas used in this study. Accordingly, the values of the critical pressure (*P*_*c*_), critical temperature (*T*_*c*_) and diameter of the methane molecule are 4.6 MPa, 190.56 K, and 0.3758 nm, respectively.

Based on Equation 7, the fugacity of the bulk fluid can be expressed as:

11 $$\mathrm{ln}\,\frac{{f}_{{\rm{bulk}}}}{p}=\frac{b\rho }{1-b\rho }-\frac{a(T)\rho }{RT(1+2b\rho -{b}^{2}{\rho }^{2})}-\,\mathrm{ln}\,[\frac{p}{RT\rho }-\frac{pb}{RT}]-\frac{a(T)}{2\sqrt{2}bRT}\,\mathrm{ln}[\frac{1+(1+\sqrt{2})\rho b}{1+(1-\sqrt{2})\rho b}]$$

Analogously, the fugacity of the adsorbate, which accounts for the fluid-fluid interactions, can be given as:

12 $$\begin{array}{c}\mathrm{ln}\,\frac{{f}_{{\rm{ff}}}(z)}{p}=\frac{{b}_{{\rm{ads}}}\rho (z)}{1-{b}_{{\rm{ads}}}\rho (z)}-\frac{{a}_{{\rm{ads}}}(z)\rho (z)}{RT(1+2{b}_{{\rm{ads}}}\rho (z)-{b}_{{\rm{ads}}}^{2}{\rho }^{2}(z))}-\,\mathrm{ln}\,[\frac{p}{RT\rho (z)}-\frac{p{b}_{{\rm{ads}}}}{RT}]\\ \quad \quad \quad \quad \,\,-\frac{{a}_{{\rm{ads}}}(z)}{2\sqrt{2}{b}_{{\rm{ads}}}RT}\,\mathrm{ln}[\frac{1+(1+\sqrt{2})\rho (z){b}_{{\rm{ads}}}}{1+(1-\sqrt{2})\rho (z){b}_{{\rm{ads}}}}]\end{array}$$

*ρ(z)* in Equation 12 is the density profile in the slit pore; thus, based on the SLD-PR model, the Gibbs adsorption isotherm can be given as:

13 $${n}^{{\rm{Gibbs}}}=\frac{A}{2}{\int }_{\frac{{\sigma }_{{\rm{ff}}}}{2}}^{L-\frac{{\sigma }_{{\rm{ff}}}}{2}}(\rho (z)-{\rho }_{{\rm{bulk}}})dz$$

In Equation 13, parameter *A* is the specific surface area (in m^2^/g), and *n*_Gibbs_ is the Gibbs adsorption amount (in mmol/g). Following Simpson’s rule, Equation 13 can be integrated numerically. To fit the methane adsorption isotherm with the SLD-PR model, the regression process will determine four parameters: specific surface area, *A* (in m^2^/g); fluid-solid interaction energy, *ε*_fs_/*k* (in Kelvin); slit width, *L* (in nm); and covolume correction factor, *A*_*b*_. Note that *ε*_fs_/*k* is the fluid-solid interaction normalized by Boltzmann’s constant (*k*), the units of which are in Kelvin.

Furthermore, following the method proposed by Pang *et al*.^34^, the thickness of the adsorbed methane can be determined. First, calculate the average value of the density at *σ*_ff_*/2* and the density at *L/2*, as denoted by *ρ*_1*/*2_*(z)*, which can be expressed as *ρ*_*1/2*_*(z)* = *(ρ*_*σ*ff*/2*_ + *ρ*_*L/2*_*)/2*. Second, compute the corresponding *z* of *ρ*_*1/2*_*(z)* from the density profile. The estimated z is regarded as the effective thickness of the adsorbed gas, from which the volume of the adsorbed gas can be determined by multiplying the surface area by the estimated z. For more details about the SLD-PR model and the thickness of the adsorbed gas, please refer to our previous work^34^.

### Stress-Dependence Effect

The stress-dependence effect can be described by the pore compressibility, as shown in the following equation:

14 $${C}_{p}=\frac{1}{{V}_{p}}{(\frac{\partial {V}_{p}}{\partial {P}_{p}})}_{{P}_{c}}=\frac{1}{\varphi }{(\frac{\partial \varphi }{\partial {P}_{p}})}_{{P}_{c}}$$

This equation indicates that the confining pressure (*P*_*c*_) is assumed to be constant under reservoir conditions. As a result, the pore volume will decrease as the pore pressure (*P*_*p*_) decreases. After simplification, Equation 14 can be expressed as follows:

15 $$\frac{\varphi }{{\varphi }_{0}}=1+{C}_{p}({P}_{p}-{P}_{p0})$$

Under reservoir conditions, pore pressure decreases during gas production, and compression caused by overburden stress leads to pore volume shrinkage due to the decrease in effective stress. *ϕ/ϕ*_*o*_ in Equation 15 is defined as the porosity multiplier. *P*_*p*_ and *P*_*po*_ are the pore pressures that correspond to *ϕ* and *ϕ*_*o*_, respectively.

### Non-Darcy Flow Effect

The gas transport mechanism in shale gas reservoirs is significantly different than that in conventional reservoirs. Darcy’s law may fail to properly describe the flow behavior in nanopores. Generally, the process of determining the flow regime based on the Knudsen number (*Kn*) and then selecting an appropriate model to characterize the flow behavior has been widely acknowledged. The Knudsen number (*Kn*) is defined as the ratio of the gas mean free path to the characteristic length.

16 $$Kn=\frac{\lambda }{D}$$

where *D* is the characteristic length and λ is the mean free path, which is calculated as follows^24^:

17 $$\lambda =\frac{kT}{\sqrt{2}\pi {\delta }^{2}P}$$

where *k* is the Boltzmann constant, *T* is the temperature in Kelvin, *δ* is the diameter of gas molecules in m, and *P* is the pressure in Pa. It is worth mentioning that when considering the gas adsorption effect, the characteristic length of the flow conduit should be corrected. Based on the slit pore geometry of the SLD-PR model, the characteristic length (*D*) in Equation 16 should be the effective slit pore width (*L*_*b*_) for free gas, which is given as:

18 $${L}_{b}=L-2z$$

where *L* is the slit pore width and *z* is the thickness of the adsorbed gas.

Based on previous studies, the flow regimes in micro- and nanoscale systems, such as shale gas reservoirs, are mainly slip flow and transition flow regimes^19,40,41^. Therefore, gas slippage and Knudsen diffusion should be considered as the primary gas flow models. Herein, taking advantage of the slit pore geometry of the SLD-PR model, we assume that all pores in the shale are slit-shaped pores. Accordingly, the gas slippage and Knudsen diffusion models with slit-shaped pores can be generated separately.

First, in terms of gas slippage, the Navier-Stokes (N-S) equation is widely used to interpret the gas flow behavior in slip flow regimes^42–44^. To extend the N-S solution to the transition flow regime, the Maxwell-type second-order slip boundary condition is used to solve the N-S equation. The general form of the second-order slip boundary condition is written as^42^:

19 $${u}_{s}=\pm {A}_{1}\lambda \frac{\partial u}{\partial y}-{A}_{2}{\lambda }^{2}\frac{{\partial }^{2}u}{\partial {y}^{2}}$$

For slit-shaped pore geometry, the characteristic length (*D*) in Equation 16 should be the effective slit width (*L*_*b*_). Subsequently, the mass flux of the second-order slip flow is given as:

20 $${J}_{vs}=\frac{{L}_{b}^{2}{\rho }_{g}}{12\mu }(-\frac{dp}{dx})(1+6{A}_{1}Kn+12{A}_{2}K{n}^{2})$$

in which

21 $${A}_{1}=\frac{2-\sigma }{\sigma }\,$$

where *A*_*1*_ and *A*_*2*_ are slip coefficients, and σ is the tangential momentum accommodation coefficient (TMAC). The tortuosity (*τ*) is set as 5 in this study, and *k*_*D*_ is the dimensionless permeability. The viscosity *µ* (in Pa·s) is calculated using the model of Lee, Gonzales and Eakin^45^.

On the other hand, the mass flux of a Knudsen diffusion slit-shaped pore can be expressed as:

22 $${J}_{k}=\frac{{D}_{k-{\rm{eff}}}}{RT}(-\frac{dp}{dx})=\frac{4L}{3\pi }{\delta }^{{D}_{f}-2}{(\frac{8}{\pi MRT})}^{0.5}(-\frac{dp}{dx})\,$$

where

23 $${D}_{k-{\rm{eff}}}={\delta }^{{D}_{f}-2}{D}_{k}$$

24 $${D}_{k}=\frac{1}{3}s{(\frac{8RT}{\pi M})}^{0.5}$$

25 $$\delta =\frac{{d}_{m}}{{L}_{b}}$$

in which *D*_*f*_ is the fractal dimension of the pore wall and *s* is the characteristic length. For slit pore geometry, *s* is given as^46^:

26 $$s=\frac{4{L}_{b}}{\pi }$$

Furthermore, in terms of adsorptive gas, such as *CH*_4_ and *CO*_2_, the effect of surface diffusion may provide extra flow velocity to enhance the free gas transport in nanopores^19,47^. Therefore, to accurately describe the adsorptive gas flow in shale gas reservoirs, surface diffusion should be considered. The driving force of surface diffusion is the chemical potential gradient. The mass flux due to surface diffusion can be calculated as^17,18^:

27 $${J}_{k}={D}_{s}\frac{{C}_{s}}{p}(-\frac{dp}{dx})$$

28 $${D}_{s}=\frac{{D}_{s0}}{1-\theta }$$

where *D*_*s*_ is the surface diffusivity in m^2^/s, *D*_*s*0_ is the surface diffusivity at zero gas coverage in m^2^/s, and *C*_*s*_ is the adsorbed phase concentration in mol/m^3^. The adsorbed phase concentration follows the Langmuir isotherm equilibrium, which is expressed as:

29 $$\theta =\frac{{n}_{a}}{{n}_{0}}=\frac{p}{p+{p}_{L}}=\frac{{C}_{s}}{{C}_{\mu s}}$$

where *θ* is the adsorption coverage, *n*_*a*_ is the amount of the adsorbed phase per mass of adsorbent, *n*_0_ is the maximum adsorption amount corresponding to the adsorbent capacity (in mmol/g), *P*_*L*_ is the Langmuir pressure (in MPa), and *C*_*µs*_ is the maximum mass of gas adsorbed per solid volume (in mol/m^3^).

Finally, following the model proposed by Wu *et al*.^7^ for gas transport in shale nanopores, the total mass flux can be written as:

30 $${J}_{t}={\omega }_{vs}{J}_{vs}+{\omega }_{k}{J}_{k}+{J}_{s}$$

where *J*_*t*_ is the total mass flux. *J*_*vs*_ and *J*_*k*_ are the mass fluxes of second-order slip flow and Knudsen diffusion, respectively. ω_vs_ is the ratio of the intermolecular collision frequency to the total collision frequency, which is expressed as^48^:

31 $${\omega }_{vs}=\frac{1}{1+\frac{Kn}{2}}$$

In addition, *ω*_*k*_ is the ratio of the wall-molecule collision frequency to the total collision frequency, which is expressed as^48^:

32 $${\omega }_{k}=\frac{1}{1+\frac{2}{Kn}}$$

Finally, the total apparent permeability of the slit-shaped nanopores can be calculated as:

33 $${k}_{t}=\frac{1}{1+\frac{Kn}{2}}\frac{{L}_{b}^{2}}{12}(1+6{A}_{1}Kn+12{A}_{2}K{n}^{2})+\,\frac{1}{1+\frac{2}{Kn}}\frac{4{L}_{b}}{3\pi }{\delta }^{{D}_{f}-2}{(\frac{8RT}{\pi M})}^{0.5}\frac{\mu }{p}+\frac{{D}_{s}{C}_{s}RT\mu }{{p}^{2}}$$

where *k*_*t*_ is the total apparent permeability (in m^2^) of the slit-shaped nanopores.

## Results and Discussion

To properly evaluate the application of the apparent permeability model to shale gas reservoirs, the apparent permeability model, which includes the effects of gas adsorption, stress dependence, and non-Darcy flow, is used to perform the regression analysis of the measured matrix permeabilities of the Barnett and Eagle Ford shale core samples.

**Figure 5:**
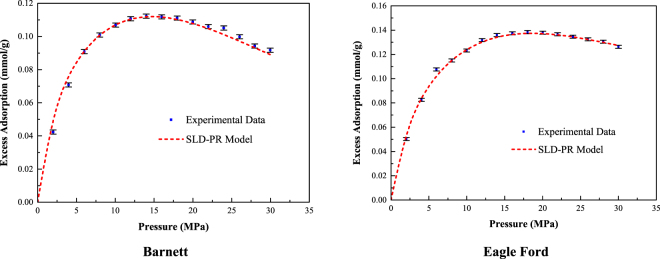
Excess methane adsorption and curve-fitting results of the Barnett and Eagle Ford shale core samples obtained using the SLD-PR model.

First, the effect of gas adsorption leads to the reduction of the effective pore width and additional gas mobility due to surface diffusion. Herein, the methane adsorption capacity of the Barnett and Eagle Ford shale core samples is evaluated by fitting the measured excess adsorption data with the SLD-PR model. Based on the pore size distribution, the mode of the pore widths obtained from the N_2_ adsorption/desorption isotherms for both the Barnett and Eagle Ford shales is approximately 3.7 to 4.2 nm. Therefore, the pore width range in the SLD-PR model is set from 3.5 to 4.5 nm to represent the methane adsorption in the majority of nanoscale pores in these shale core samples. The four regression parameters and the average absolute percent error (*%AAD*) of the SLD-PR model are presented in Table 4. The function of the average absolute percent error (*%AAD*) is given in Appendix B. In addition, the measured excess methane adsorption isotherms and the curve-fitting results are shown in Fig. 5.

**Table 4:** Curve-fitting parameters and %AAD of the SLD-PR model for the Barnett shale core samples.

Core Sample	A (m^2^/g)	ε_fs_/k (K)	L (nm)	A_b_	%AAD
Barnett	27.8	75.8	3.7	0.05	3.4591
Eagle Ford	32.2	70.6	4.2	0.00	1.9550

The best match (%AAD = 3.4591) of the slit-shaped pore width for the Barnett shale core sample is 3.7 nm, and the best match (%AAD = 1.955) of the slit-shaped pore width for the Eagle Ford shale core sample is 4.2 nm. These values are used as the pore widths (*L*) in the following calculations. The pore volume of the Barnett shale core samples can be calculated by multiplying the surface area by half the pore width due to the slit-shaped pore geometry; the corresponding porosity is 0.1185. Similarly, the porosity of the Eagle Ford shale core sample is 0.1574.

Furthermore, according to the density profile provided by the SLD-PR model, the thickness of the adsorbed gas can be determined using the method proposed by Pang *et al*.^34^. The thickness of the adsorbed methane (*z*) is presented in Table 5. Subsequently, the volume of the adsorbed methane can be calculated by multiplying the surface area by the thickness. Then, the absolute adsorption is calculated as follows^49^:

34 $${n}_{{\rm{ads}}}^{{\rm{Gibbs}}}={n}_{{\rm{ads}}}^{{\rm{Abs}}}-{V}_{{\rm{ads}}}\cdot {\rho }_{{\rm{gas}}}$$

**Table 5:** Calculation results of the Barnett shale for the combination of gas adsorption and stress-dependence effects.

Pore Pressure (MPa)	Bulk Gas Density (kg/m^3^)	Adsorption Thickness (nm)	Volume of Adsorption (cm^3^)	Density of Adsorption (kg/m^3^)	Absolute Adsorption (mmol/g)	Porosity Multiplier	PV (cm^3^)	Effective PV (cm^3^)	Effective Porosity Multiplier
5.08	29.0056	0.2983	1.6601	194.6423	0.1009	0.9908	10.2005	8.5405	0.8295
6.05	34.8257	0.3051	1.6979	208.4213	0.1105	0.9923	10.2159	8.5180	0.8274
7.05	40.8921	0.3113	1.7324	220.6441	0.1193	0.9938	10.2319	8.4995	0.8256
8.07	47.1345	0.3169	1.7636	231.4975	0.1275	0.9954	10.2480	8.4845	0.8241
9.05	53.1693	0.3217	1.7903	240.6712	0.1345	0.9969	10.2636	8.4733	0.8230
10.04	59.2879	0.3260	1.8142	248.9187	0.1410	0.9985	10.2794	8.4652	0.8222
11.04	65.4758	0.3298	1.8354	256.3856	0.1469	1.0000	10.2953	8.4600	0.8217

PV: pore volume.

Finally, the density of the adsorbed methane is computed using:

35 $${\rho }_{{\rm{ads}}}=\frac{{n}_{{\rm{ads}}}^{{\rm{Abs}}}}{{V}_{{\rm{ads}}}}$$

Second, as shown in Fig. 4, the red trend lines indicate that there is a linear relationship between pore volume and pore pressure. Therefore, the pore compressibility (*C*_*p*_) of the Barnett shale core sample and that of the Eagle Ford shale core sample are calculated using Equation 14 to be 0.001552 1/MPa and 0.001363 1/MPa, respectively. Then, the porosity multipliers and thus the pore volumes (PVs) of both shale core samples can be calculated.

According to Equation 15, the pore volume is pore-pressure-dependent. Additionally, the adsorption amount is pore-pressure-dependent, which indicates that the volume of adsorbed gas is also pore-pressure-dependent. Hence, the stress-dependence and gas adsorption effects can be combined to describe the effective pore volume:

36 $${V}_{p({\rm{eff}})}(p)={V}_{p(s-d)}(p)-{V}_{{\rm{ads}}}(p)$$

Then, the effective pore volumes and the effective porosity multipliers of the Barnett and Eagle Ford shale core samples can be easily determined. The calculation results of the Barnett and Eagle Ford shales are summarized in Tables 5 and 6, respectively.

**Table 6:** Calculation results of the Eagle Ford shale for the combination of gas adsorption and stress-dependence effects.

Pore Pressure (MPa)	Bulk Gas Density (kg/m^3^)	Adsorption Thickness (nm)	Volume of Adsorption (cm^3^)	Density of Adsorption (kg/m^3^)	Absolute Adsorption (mmol/g)	Porosity Multiplier	PV (cm^3^)	Effective PV (cm^3^)	Effective Porosity Multiplier
5.06	28.8864	0.2938	1.9124	190.1237	0.1124	0.9906	13.5413	11.6289	0.8507
6.05	34.8257	0.3003	1.9551	205.7053	0.1243	0.9919	13.5596	11.6046	0.8489
7.03	40.7702	0.3061	1.9924	219.2123	0.1350	0.9932	13.5778	11.5854	0.8475
8.04	46.9502	0.3113	2.0263	231.6079	0.1451	0.9946	13.5965	11.5702	0.8464
9.03	53.0458	0.3158	2.0555	242.5588	0.1541	0.9959	13.6148	11.5593	0.8456
10.03	59.2260	0.3198	2.0816	252.6244	0.1626	0.9973	13.6334	11.5518	0.8450
11.02	65.3521	0.3233	2.1043	261.7497	0.1703	0.9987	13.6518	11.5475	0.8447
12.01	71.4710	0.3263	2.1244	270.1477	0.1774	1.0000	13.6703	11.5459	0.8446

PV: pore volume.

Furthermore, to perform the regression analysis of the measured permeabilities of the Barnett and Eagle Ford shale core samples using the apparent permeability model, Equation 32 should be updated with the correction factors (*ζ*_*mb*_ and *ζ*_*ms*_), as follows:

37 $${k}_{t}=\frac{1}{1+\frac{Kn}{2}}{\zeta }_{{\rm{mb}}}\frac{{L}_{b}^{2}}{12}(1+6{A}_{1}Kn+12{A}_{2}K{n}^{2})+\,\frac{1}{1+\frac{2}{Kn}}{\zeta }_{{\rm{mb}}}\frac{4{L}_{b}}{3\pi }{\delta }^{{D}_{f}-2}{(\frac{8RT}{\pi M})}^{0.5}\frac{\mu }{p}+\frac{{\zeta }_{{\rm{ms}}}{D}_{s}{C}_{s}RT\mu }{{p}^{2}}$$

where

38 $${\zeta }_{{\rm{mb}}}=\frac{{\varphi }_{tb}}{\tau }$$

39 $${\zeta }_{{\rm{ms}}}=\frac{{\varphi }_{t}-{\varphi }_{tb}}{\tau }=\frac{{\varphi }_{ts}}{\tau }$$

in which *τ* is the tortuosity, *ϕ*_*tb*_ is the total porosity for bulk free gas, *ϕ*_*t*_ is the total porosity, and *ϕ*_*ts*_ is the total porosity for surface diffusion. The derivation of the correction factors is shown in Appendix C. Moreover, compared with the permeability of the slit pores based on Darcy’s law without considering the effects of gas adsorption, stress dependence, and non-Darcy flow shown as Equation 40, the apparent permeability model (Equation 37) is more complicated due to the consideration of unique shale features.

40 $${k}_{{\rm{Darcy}}}=\frac{{L}^{2}}{12}\frac{{\varphi }_{t}}{\tau }$$

In this study, the total porosity (*ϕ*_*t*_) is calculated based on the PV values in Tables 5 and 6, and the total porosity for bulk free gas (*ϕ*_*tb*_) is calculated based on the effective PV values in Tables 5 and 6. In addition, by fitting the absolute adsorption amounts shown in Tables 5 and 6 with the Langmuir isotherm (Equation 29), the Langmuir pressure (*P*_*L*_), maximum adsorption amount (*n*_*0*_), and maximum mass of gas adsorbed per solid volume (*C*_*µs*_) for the Barnett shale are determined to be 6.513 MPa, 0.2311 mmol/g, and 9.6433 kg/m^3^, respectively. In addition, these factors for the Eagle Ford shale are 8.964 MPa, 0.3084 mmol/g, and 13.6239 kg/m^3^, respectively. Finally, the adsorbed phase concentration (*C*_*s*_) can easily be obtained.

**Figure 6:**
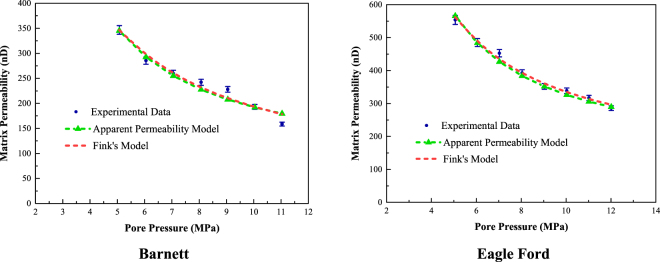
Curve-fitting results of matrix permeability versus pore pressure for the Barnett and Eagle Ford shale core samples.

Regression analysis can be used to determine four critical parameters: the first-order and second-order slip coefficients (*A*_1_ and *A*_2_), tortuosity (*τ*), and surface diffusivity at zero coverage (*D*_*s0*_). According to previous studies^40,41,46,50^, the reliable ranges of these four parameters are as follows: *A*_1_ - from 0.5 to 2; *A*_2_ - from 0.2 to 1.2; *τ* - from 2.3 to 11.9; and *D*_*s*0_ - from 1 × 10^−8^ to 1 × 10^−4^ m^2^/s. The four regression parameters and average absolute percent error (*%AAD*) values are exhibited in Table 7. In addition, the measured permeabilities and curve-fitting results are displayed in Fig. 6. The low %AAD values and curve-fitting results presented in Fig. 6 may indicate that the apparent permeability model can appropriately evaluate the permeability of shale gas reservoirs. To further verify the applicability of the developed apparent permeability model, Fink’s permeability model, which is expressed as a superposition of slip flow considering the Klinkenberg effect and the pore-elastic effect, is used as a comparison. Fink’s permeability model is given as^24^:

41 $${k}_{{\rm{gas}}}={k}_{\infty ,0}{e}^{{\alpha }_{k}({P}_{c}-\chi {P}_{p})}\cdot [1+\frac{{b}_{0}+\beta ({P}_{c}-\chi {P}_{p})}{{P}_{p}}]$$

where *k*_*∞,0*_ is the Klinkenberg-corrected permeability coefficient at zero effective stress, *α*_*k*_ is an adjustable parameter indicating stress sensitivity, *χ* determines the relative sensitivity of permeability to changes in pore pressure, *b*_*0*_ is the gas slippage factor at zero effective stress, and *β* is the slope of the linear best fit that indicates stress sensitivity. The curve-fitting results and regression parameters are shown in Fig. 6 and Table 8, respectively. From the curve-fitting results of the developed apparent permeability model and Fink’s model shown in Fig. 6, it is obvious that both permeability models can properly describe changes in the permeability of the shale matrix with pore pressure. However, compared with Fink’s model, the apparent permeability model proposed in this study has several advantages. First, based on the Knudsen number, the second-order gas slippage and Knudsen diffusion are considered to extend the applicability of this model to the transition flow regime (0.18 < *Kn* < 0.5). Second, the gas adsorption effect, which includes the reduction in pore width and surface diffusion, is incorporated to represent the flow behavior of adsorptive gas (methane) in nanopores. In addition, surface diffusivity, which is difficult to measure in the laboratory, can be obtained via the regression analysis of the measured permeability of shale rocks. In this study, the determined surface diffusivity is on the order of 10^−5^, which is consistent with published data^19,50^.

**Table 7:** Regression parameters and %AAD of permeability curve-fitting using the apparent permeability model.

Core Sample	A_1_	A_2_	τ	D_s0_ (m^2^/s)	%AAD
Barnett	1.47	0.78	2.32	1.98 × 10^−5^	4.7749
Eagle Ford	1.59	0.47	2.30	3.00 × 10^−5^	2.3284

**Table 8:** Regression parameters and %AAD of permeability curve-fitting using Fink’s model.

Core Sample	k_∞,0_ (nD)	α_k_ (MPa^−1^)	χ	b_0_ (MPa)	β (MPa^−1^)	%AAD
Barnett	125.9	−0.0001	0.8972	0.5238	0.8011	4.2210
Eagle Ford	213.1	−0.0001	0.7353	0.3239	0.7066	1.8162

Moreover, the Knudsen numbers and permeabilities attributed to the different gas transport phenomena of the Barnett and Eagle Ford shales are shown in Tables 9 and 10, respectively.

**Table 9:** Knudsen numbers and permeabilities due to different gas transport phenomena of the Barnett shale.

Pore Pressure (MPa)	Knudsen Number	Permeability of Gas Slippage (nD)	Permeability of Knudsen Diffusion (nD)	Permeability of Surface Diffusion (nD)	Apparent Permeability (nD)
5.08	0.4961	212.0433	7.5009	125.4894	345.0335
6.05	0.4184	178.3189	5.5336	108.5322	292.3847
7.05	0.3605	154.1796	4.2394	96.5405	254.9596
8.07	0.3161	136.2980	3.3531	87.8488	227.4999
9.05	0.2828	123.2678	2.7515	81.6495	207.6688
10.04	0.2556	112.9347	2.3056	76.9235	192.1638
11.04	0.2330	104.5679	1.9612	73.0527	179.5818

**Table 10:** Knudsen numbers and permeabilities due to different gas transport phenomena of the Eagle Ford shale.

Pore Pressure (MPa)	Knudsen Number	Permeability of Gas Slippage (nD)	Permeability of Knudsen Diffusion (nD)	Permeability of Surface Diffusion (nD)	Apparent Permeability (nD)
5.06	0.4279	323.0326	9.8923	232.8697	565.7946
6.05	0.3592	277.6226	7.2227	198.9411	483.7864
7.03	0.3101	245.5701	5.5460	175.8046	426.9207
8.04	0.2719	220.9662	4.3835	158.5151	383.8648
9.03	0.2427	202.3524	3.5813	145.8241	351.7578
10.03	0.2190	187.4072	2.9876	135.9238	326.3186
11.02	0.1997	175.3785	2.5437	128.1873	306.1096
12.01	0.1836	165.4312	2.1996	121.9219	289.5528

Based on their Knudsen numbers, the flow regimes for both the Barnett and Eagle Ford shale core samples represent the transition flow regime. However, their Knudsen numbers are far less than 1 (0.18 < *Kn* < 0.5); thus, the permeability of Knudsen diffusion contributes little to the total apparent permeability, as Knudsen diffusion will dominate the flow behavior when *Kn* ≥ 1^9,17^. In contrast, if the Knudsen number is much larger than 1, Knudsen diffusion may account for a larger proportion of the total apparent permeability. Moreover, Equation 37 indicates that Knudsen diffusion is only dependent on the Knudsen number (*Kn*) and tortuosity (*τ*). Thus, only the pore size distribution and the thickness of adsorbed methane can cause variations in *Kn* and thus lead to a significant difference. Compared with Knudsen diffusion, gas slippage and surface diffusion contribute the most to the total apparent permeability. The contribution from surface diffusion due to gas adsorption accounts for approximately 50% of the total apparent permeability. Therefore, the gas adsorption effect plays an important role in characterizing the shale permeability. In addition, the permeabilities of both gas slippage and surface diffusion increase as the pore pressure decreases, which indicates that the gas flow in shale gas reservoirs may be enhanced during gas production. This finding may deserve close attention from production engineers.

## Conclusions

In this study, an apparent permeability model is developed to characterize the gas flow behavior in nanoporous shale. This model considers the effects of gas adsorption, stress dependence, and surface diffusion. Here, experimental measurements of the gas adsorption/desorption, stress-dependent pore volume, and matrix permeability of shale were conducted; then, multiparameter analysis of the measured data was performed to determine the critical parameters contributing to gas flow in nanopores. The experimental studies and multiparameter analysis yielded the following conclusions.

1. The SLD-PR model can properly evaluate the excess methane adsorption capacity of the Barnett and Eagle Ford shales measured in the laboratory. By considering the pore size distribution obtained from nitrogen adsorption and desorption isotherms, this model can reliably estimate the effective slit-shaped pore width in order to calculate the Knudsen number.
2. Gas adsorption has two competitive effects. Adsorbed gas will reduce the effective pore width for gas flow in nanopores, while surface diffusion will provide additional mobility to enhance gas flow in nanopores. The reduction in pore width leads to a larger Knudsen number, which has an impact on the flow regime determination. Meanwhile, the movement of adsorbed gas due to surface diffusion largely contributes to the apparent permeability of shale.
3. The developed apparent permeability model, which considers the effects of gas adsorption, stress dependence, and non-Darcy flow, can appropriately characterize the shale permeability measured using the pressure-pulse decay method.
4. The non-Darcy flow effect, which is expressed as the combination of second-order slippage, Knudsen diffusion, and surface diffusion, enables researchers to describe the gas flow behavior in the transition flow regime. In this study, gas slippage and surface diffusion dominate the gas flow in nanopores, with Knudsen numbers ranging from 0.18 to 0.5.
5. Within a given reliable and reasonable range, the slip coefficients, tortuosity, and surface diffusivity, which are difficult to measure in the laboratory, can be predicted by fitting the measured permeability with the apparent permeability model.

## Electronic supplementary material

Appendix
