# Supplementary material for: Multiparameter Analysis of Gas Transport Phenomena in Shale Gas Reservoirs: Apparent Permeability Characterization
Source: Sci Rep. 2018 Feb 8;8:2601. doi: 10.1038/s41598-018-20949-2 (PMC5805773; doi:10.1038/s41598-018-20949-2)
Supplement: Supplementary file 1 — Appendix [file 41598_2018_20949_MOESM1_ESM.pdf]

Multiparameter Analysis of Gas Transport Phenomena in Shale Gas Reservoirs: Apparent Permeability Characterization

Yinghao Shen [1], Yu Pang [2*], Ziqi Shen [2], Yuanyuan Tian [3] Hongkui Ge [1 4]

1. Unconventional Natural Gas Institute, China University of Petroleum, Beijing 102249, China. 2. Department of Petroleum Engineering, Texas Tech University, Lubbock, TX 79409, USA. 3. State Key Laboratory for Oil and Gas Reservoir Geology and Exploitation (Chengdu University of Technology) Chengdu 610059, China. 4. China University of Petroleum-Beijing at Karamay

Appendix A

The experimental devices of gas expansion method are displayed as follows.

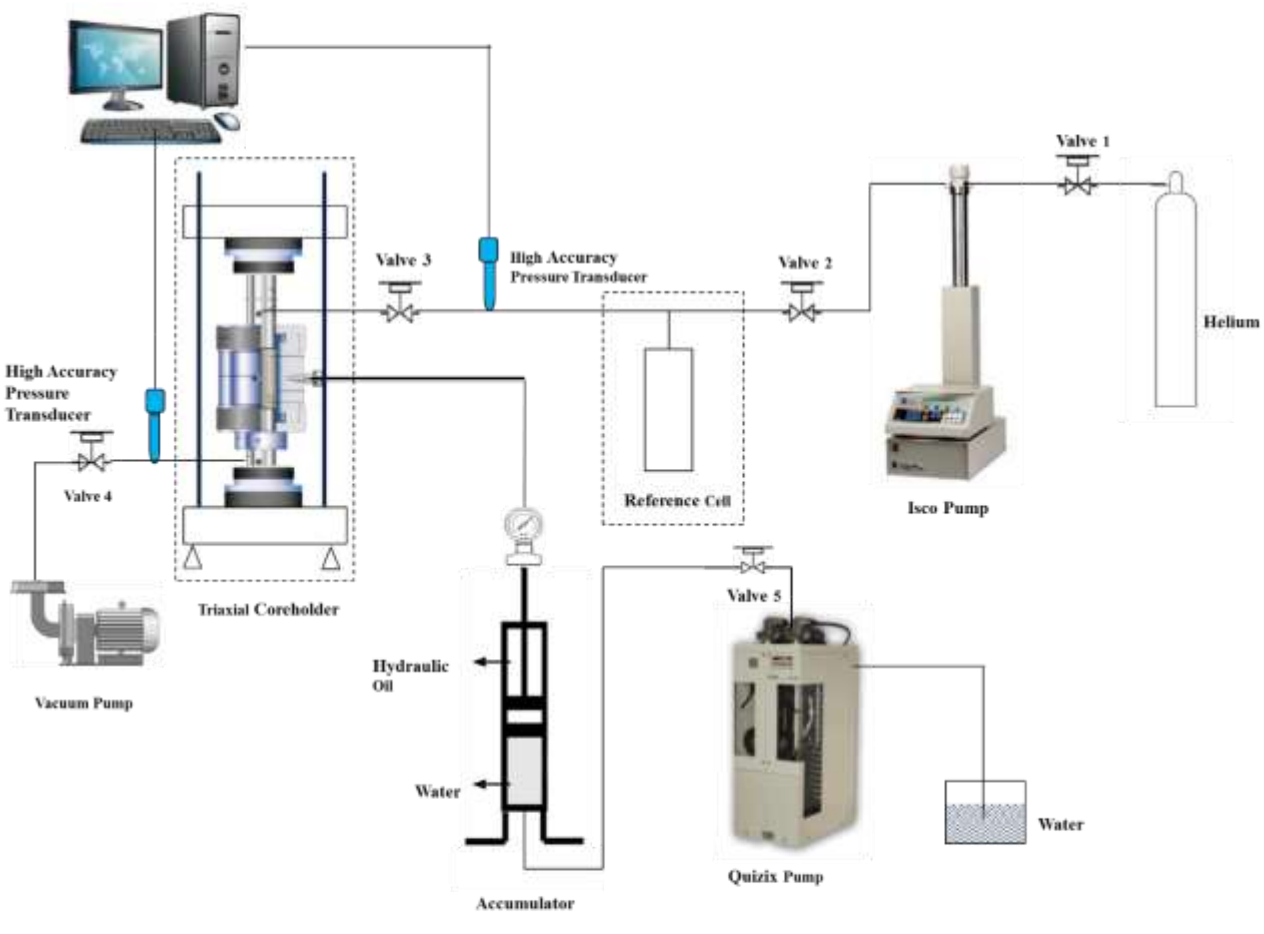

Figure A-1 Schematic diagram of pore compressibility measurement devices utilizing the gas expansion method

Appendix B

The expression of average absolute percent error (*%AAD*) is given as follow:

$$\%AAD = \frac{1}{N}\sum_{i=1}^{N}\left|\frac{n_{calc}-n_{exp}}{n_{exp}}\right| \times 100$$

where N is the number of data point. n_{calc} is the value calculated by theoretical model and n_{exp} is the value measured from laboratory experiments.

Appendix C

As the geometry Figure (C-1) illustrated, several slit pores are in a rock formation with an area (A_t).

For bulk phase (free gas), the total bulk flow rate can be written as:

$$q_{tb} = \frac{nk_bA_b}{\mu}\frac{\Delta p}{L}$$

$$q_{tb} = \frac{k_{tb}A_t}{\mu}\frac{\Delta p}{L'}$$

After rearrangement,

$$\frac{nk_bA_b}{k_{tb}A_t} = \frac{L}{L'}$$

$$n = \frac{A_tL'\phi_{tb}}{A_bL}$$

$$\frac{k_b\phi_{tb}}{k_{tb}} = \left(\frac{L}{L'}\right)^2$$

Tortuosity is defined as:

$$\tau = \left(\frac{L}{L'}\right)^2$$

$$\frac{k_b\phi_{tb}}{\tau} = k_{tb}$$

Finally, the correction factor for bulk phase is given as:

$$\zeta_{mb} = \frac{\phi_{tb}}{\tau}$$

Similarly, for adsorbed gas (surface diffusion)

$$q_{ts} = \frac{nk_sA_b}{\mu}\frac{\Delta p}{L}$$

$$q_{ts} = \frac{k_{ts}A_t}{\mu}\frac{\Delta p}{L'}$$

$$\frac{nk_sA_s}{k_{ts}A_t} = \frac{L}{L'}$$

$$n = \frac{A_tL'\phi_{ts}}{A_sL}$$

Total porosity is the sum of porosity for bulk free gas and porosity occupied by adsorbed gas.

$$\phi_{ts} + \phi_{tb} = \phi_t$$

$$\frac{k_s \phi_{ts}}{k_{ts}} = \left(\frac{L}{L'}\right)^2$$

$$\frac{k_s(\phi_t - \phi_{tb})}{k_{ts}} = \left(\frac{L}{L'}\right)^2$$

Finally, the correction factor for adsorbed phase is given as:

$$\frac{k_s(\phi_t - \phi_{tb})}{\tau} = k_{ts}$$

$$\zeta_{ms} = \frac{\phi_t - \phi_{tb}}{\tau}$$

$$A_t = w_m h_m$$

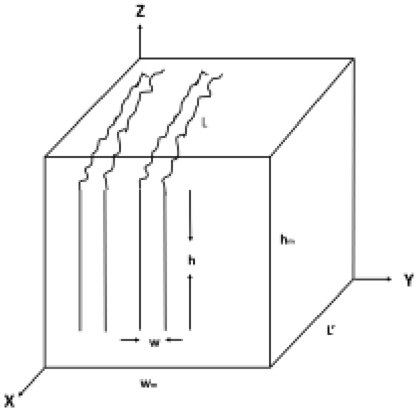

Figure C-1. Formation with several slit-shaped pores
